# Oviposition of *Aedes japonicus japonicus* (Diptera: Culicidae) and associated native species in relation to season, temperature and land use in western Germany

**DOI:** 10.1186/s13071-020-04461-z

**Published:** 2020-12-17

**Authors:** Linus Früh, Helge Kampen, Marcel B. Koban, Nadja Pernat, Günter A. Schaub, Doreen Werner

**Affiliations:** 1 Leibniz Centre for Agricultural Landscape Research, Eberswalder Straße 84, 15374 Müncheberg, Germany; 2 Friedrich-Loeffler-Institut, Federal Research Institute for Animal Health, Südufer 10, Insel Riems, 17493 Greifswald, Germany; 3 Universität Hohenheim, Garbenstraße 30, 70593 Stuttgart, Germany; 4 Ruhr-Universität Bochum, Universitätsstraße 150, 44801 Bochum, Germany; 5 Freie Universität Berlin, Königin-Luise-Straße 1-3, 14195 Berlin, Germany

**Keywords:** Transition zone, Oviposition, Microhabitat, Land use type, Asian bush mosquito, Asian rock pool mosquito, *Aedes japonicus japonicus*, *Culex pipiens*, *Anopheles plumbeus*, *Aedes geniculatus*

## Abstract

**Background:**

*Aedes japonicus japonicus*, first detected in Europe in 2000 and considered established in Germany 10 years later, is of medical importance due to its opportunistic biting behaviour and its potential to transmit pathogenic viruses. Its seasonal phenology, temperature and land use preference related to oviposition in newly colonised regions remain unclear, especially in the context of co-occurring native mosquito species.

**Methods:**

Focussing on regions in Germany known to be infested by *Ae. japonicus japonicus*, we installed ovitraps in different landscapes and their transition zones and recorded the oviposition activity of mosquitoes in relation to season, temperature and land use (arable land, forest, settlement) in two field seasons (May–August 2017, April–November 2018).

**Results:**

*Ae. japonicus japonicus* eggs and larvae were encountered in 2017 from June to August and in 2018 from May to November, with a markedly high abundance from June to September in rural transition zones between forest and settlement, limited to water temperatures below 30 °C. Of the three native mosquito taxa using the ovitraps, the most frequent was *Culex pipiens* s.l., whose offspring was found in high numbers from June to August at water temperatures of up to 35 °C. The third recorded species, *Anopheles plumbeus*, rarely occurred in ovitraps positioned in settlements and on arable land, but was often associated with *Ae. japonicus japonicus*. The least frequent species, *Aedes geniculatus*, was mostly found in ovitraps located in the forest.

**Conclusions:**

The transition zone between forest and settlement was demonstrated to be the preferred oviposition habitat of *Ae. japonicus japonicus*, where it was also the most frequent container-inhabiting mosquito species in this study. Compared to native taxa, *Ae. japonicus japonicus* showed an extended seasonal activity period, presumably due to tolerance of colder water temperatures. Higher water temperatures and arable land represent distribution barriers to this species. The frequently co-occurring native species *An. plumbeus* might be useful as an indicator for potentially suitable oviposition habitats of *Ae. japonicus japonicus* in hitherto uncolonised regions. The results contribute to a better understanding of mosquito ecology and provide a basis for more targeted monitoring, distribution modelling and risk management of mosquitoes.

**Graphical Abstract:**

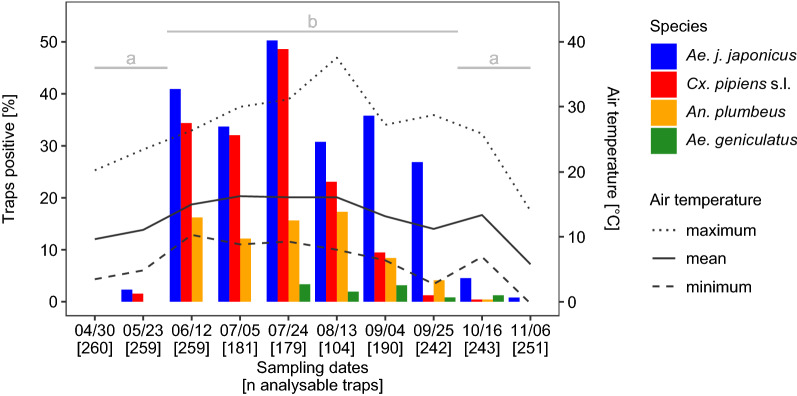

## Background

The necessity to explore the ecology of the Asian bush mosquito (Asian rock pool mosquito) *Aedes* (*Hulecoeteomyia*) *japonicus japonicus* (Theobald, 1901) is predominantly based on its possible role as a vector of pathogens, including chikungunya virus, West Nile virus (WNV), Zika virus and various other encephalitis viruses [1–5]. Due to its opportunistic biting behaviour [6], *Ae. japonicus japonicus* is a possible bridge vector, which is able to transmit avian-borne zoonotic disease agents such as WNV to mammals including humans. Its vector potential and its natural dispersal capabilities may pose serious risks to human and animal health [7, 8].

The area of origin of *Ae. japonicus japonicus* covers eastern Russia, eastern China, Korea and Japan [9, 10]. The species comprises four subspecies [9], which were recently discussed to be separate species [11]. Until now, only the subspecies *Ae. japonicus japonicus* has been demonstrated to occur outside of its native area. This subspecies started to spread in North America in the late 1990s [12] and to colonise Europe from 2000 onwards [13]. The most recent findings were reported from Spain [14], Serbia and Bosnia and Herzegovina [15]. Due to its geographic origin, this mosquito is well adapted to the moderate climate of Germany, where it has been considered established since 2008 [16]. The genetic mixing of the populations by new introduction events [17] includes the possibility of a higher adaptation capacity and is of concern [18, 19]. On the other hand, the development of larvae of *Ae. japonicus japonicus* is limited at water temperatures higher than 30 °C [20, 21]. In the USA, larval populations of the Asian bush mosquito have an advantage over those of native species like *Aedes triseriatus* (Say, 1823) and *Aedes atropalpus* (Coquillett, 1902) when developing at lower water temperatures and limited resources [22]. However, larvae of *Ae. japonicus japonicus* are more sensitive to higher water temperatures than those of native species, e.g. *Aedes atropalpus* in the USA [23], which should prevent displacement of the latter.

Several studies have investigated the distribution of *Ae. japonicus japonicus* on a regional scale in its native area [9, 24, 25], as well as outside of its area of origin [26–29]. However, knowledge gaps exist with respect to its occurrence and spread at the local level. Questions remain regarding the effect of land use types on oviposition and larval habitat quality and stability as well as population dynamics of the subspecies, particularly in relation to native mosquito taxa. As the distribution of the Asian bush mosquito is patchy in Germany, factors promoting or inhibiting its dispersal might exist at the landscape level [30, 31]. This has been documented in other countries, with *Ae. japonicus japonicus* showing a preference for rural areas in the USA [26] and avoidance of large forests (bigger than 500 ha) and large areas of arable land in Hungary [32].

Observations on mosquito oviposition and larval habitat preference conducted decades ago identified euryoecious, e.g. *Culex pipiens* s.l. and *Culiseta annulata* (Schrank, 1776), and rather specialised taxa, e.g. *Anopheles plumbeus* Stephens, 1828 and *Aedes geniculatus* (Olivier, 1791), whose larvae are primarily found in tree holes [33, 34]. As these taxa are also known to use artificial containers for oviposition [33–36] their developmental stages can be expected to be found syntopically with the Asian bush mosquito. Habitat sharing of *Ae. japonicus japonicus* larvae and pupae with native taxa, however, has not been studied before in Germany. Transitional areas between different land use types generally have a special significance for the occurrence of adult mosquitoes due to their high structural diversity [37], but were not considered in previous studies on the oviposition habitat preference of mosquito species [34, 35].

Hence, the aim of the present study was to investigate the ecology of *Ae. japonicus japonicus* and co-occurring native taxa with the focus on trapping sites selected for oviposition and larval site characteristics. In particular, we hoped to elucidate whether the seasonal mean air temperature influences oviposition phenology, and thus induces differences in the seasonal presence of mosquito taxa. Another aim of this study was to check if specific water temperature ranges regulate the occurrence of larvae of *Ae. japonicus japonicus* and/or native species. Furthermore, it was tested if land use type affects oviposition activity, resulting in different abundances of ovitraps positive for the specific mosquito taxa at and around transition zones between forest (types), arable land and settlements. Finally, we studied if water temperature, tree species, land use-dependent trap location and co-occurrence of native mosquitoes can be used in a generalised linear model as predictors for the occurrence of *Ae. japonicus japonicus*-positive oviposition traps.

More detailed information on the oviposition and larval habitat preference of *Ae. japonicus japonicus* should provide a basis for more targeted monitoring measures, occurrence and dispersal models, risk analyses and integrated control measures for this subspecies [13, 16, 38].

## Materials and methods

### Study sites

**Fig. 1:**
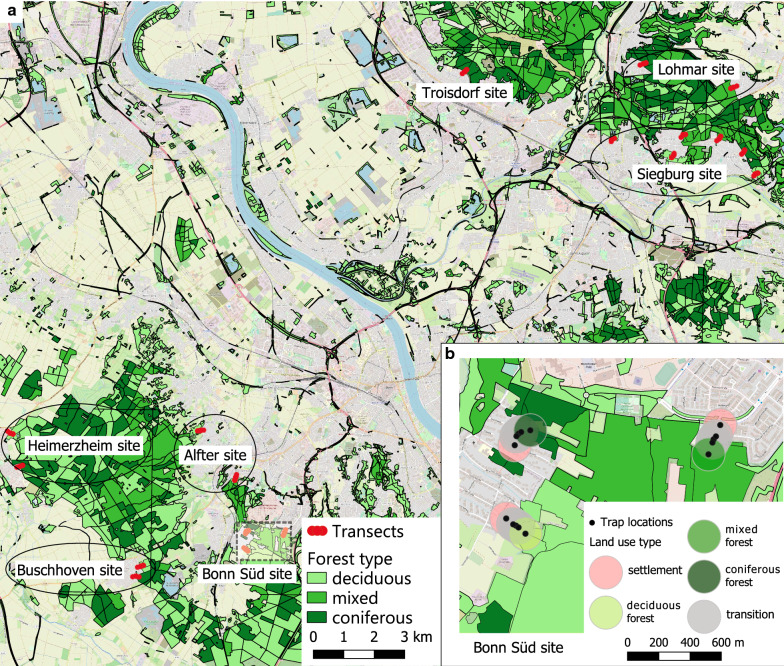
**a** Study sites in the south of North Rhine-Westphalia, Germany in 2018. Forest types (*different shades of green*) follow Authorised Topographic-Cartographic Information System data [39]. **b** Details of study site Bonn Süd, with three transects and their respective trap locations (*different colours* represent different land use types). See Additional file 2: dataset S1 for coordinates of trap locations. Background map from http://www.openstreetmap.org (OpenStreetMap contributors). The map was produced with QGIS version 3.2

The field studies were conducted in western Germany in several areas close to the river Rhine in the federal state of North Rhine-Westphalia. The 2017 study sites were located in and around the cities of Dormagen and Alfter (Additional file 1: Figs. S1 and S2). The landscape of Dormagen is shaped by arable land, settlement and commercial areas as well as by small forest patches. Alfter is almost completely surrounded by a large forest, opening only to the east to areas dominated by arable land. In 2018, the study sites were located in the Rhein-Sieg-Kreis district in the south of the federal state of North Rhine-Westphalia, specifically in Alfter, Bonn Süd, Heimerzheim, Buschhoven, Siegburg, Lohmar and Troisdorf (Fig. 1).

### Trap installation

**Fig. 2:**
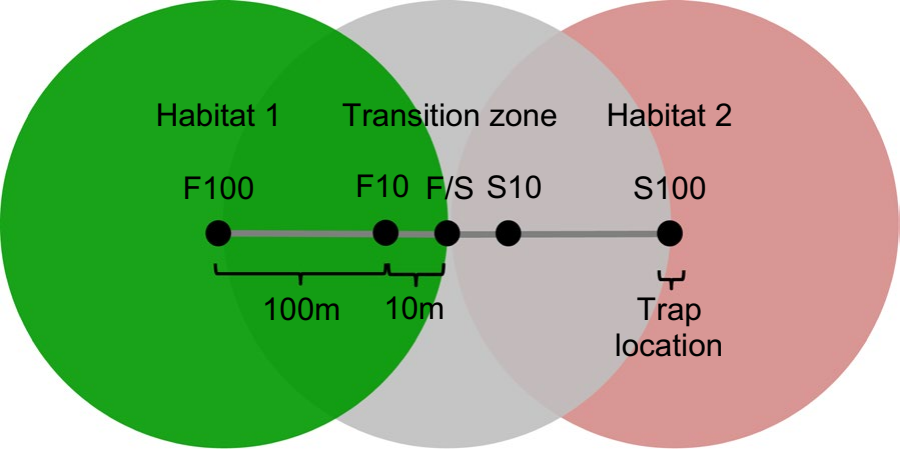
Setup of the transects. Trap locations range from oviposition habitat 1 (land use types—arable land, forest or settlement) through the transition zone into oviposition habitat 2 (land use types—forest, settlement or arable land). *F100* Forest, 100 m from the transition zone;* F10* forest, 10 m from the transition zone;* F/S* transition zone;* S10* settlement, 10 m from the transition zone;* S100* settlement, 100 m from the transition zone

In 2017, one hundred black plastic cups (400 ml volume, 12 cm high, 8 cm wide) (Bamaplast, Pieve a Nievole, Italy) and one hundred drilled logs of European beech (*Fagus sylvatica*) were distributed as possible oviposition habitats for mosquito species whose larvae can be found in artificial containers or tree holes. The beech logs [constructed from trees from a forest in Schönholz, Brandenburg (52.7950N, 13.7609E)] were approximately 20 cm long and 15 cm wide with a drilled hole 12 cm deep and eight cm wide, comparable to the dimensions of the plastic cups. The logs were sealed on the bottom with sealant (silyl modified polymer; Master-Fix; Toom, Cologne, Germany) to prevent drainage of the water. Both types of container, henceforth referred to as ‘ovitraps’, were filled with 400 ml water, collected from a nearby quarry pond in Dormagen (51.0938N, 6.7831E) and filtered through a microstrainer with a mesh size of 0.03 cm × 0.03 cm. Each plastic cup was equipped with a masonite stick as an additional oviposition substrate, the function of which was met by small cracks and crevices inside the bore hole of the wooden logs. The two types of ovitrap were placed along 20 transects; there were five trap sites per transect, and one cup and one log were placed at each site in 2017. To mitigate the effect of missing or toppled traps on the analysis of the results, three cups were set per site in 2018. Each 200 m-transect spanned two adjacent land use types, with half (100 m) of the transect in each (Fig. 2), following the method of Reiskind et al. [37].

Three different types of transects—arable land–forest, forest–settlement, and arable land–settlement—were established three times each in the same way at the study sites Dormagen (Additional file 1: Fig. S1) and Alfter (Additional file 1: Fig. S2).

In order for an area to qualify as representative of one of the required land use types, at least 90% of the area had to comprise that specific land use type (100 m radius). One transect was located in the centre of each study site (settlement–settlement), which to our knowledge did not contain, and was at least 500 m away from, natural oviposition sites (i.e. forested areas), to check if specimens of *Ae. japonicus japonicus* colonised the settlement area. Due to difficulty in finding an appropriate location for the third transect—arable land–settlement—in Dormagen, the ovitraps were placed in an urban park, resulting in two settlement–settlement transects at this site. After they were set up on 28 April, the ovitraps were examined at intervals of 3 weeks, six times in total (18 May to 31 August).

In 2018, two hundred and seventy ovitraps with masonite sticks were placed on 18 transects (Fig. 1). Based on the 2017 field study results, transects were selected with transition zones between settlement areas and three different types of forest. The forested areas were pre-selected using QGIS based on Authorative Topographic-Cartographic Information System data [39] and were included when more than 90% of the transect was covered by a specific type of tree, i.e. deciduous or coniferous. Mixed forests were defined as consisting of approximately 50:50 deciduous:coniferous tree species. Following the guidelines of the European Centre for Disease Prevention and Control [40], three ovitraps in each trapping site within the transects were filled with water, which was taken from a spring in Alfter (50.72722N, 7.00944E). After they were set up on 10 April, the ovitraps were examined every 3 weeks, ten times in total (30 April to 6 November). The species and number of trees were noted in a 10 m-radius around the individual trap sites. Air temperature was monitored hourly for all field studies using data loggers (EBI 20-TH1; Xylem Analytics, Ingolstadt, Germany), which were fixed with wire onto the stem of a shrub or trunk of a tree or on a stick at a height of approximately 30 cm, close to a trap. One data logger was placed at each trap location on three selected transects of all transect types. Maximum, minimum and mean air temperatures were calculated from hourly measurements taken in the week before sampling until the sampling date. As the data loggers on two transects produced errors, we used data from the third transect (site Bonn Süd, 50.69317N, 7.05195E). As the temperature differences between the trap locations were small, we calculated the mean from the data loggers of this transect. Water temperature was measured in each ovitrap with a digital thermometer (scaling 0.1 °C; Sainlogic, London, United Kingdom) during the process of sampling (8 a.m. until 8 p.m.).

### Sampling procedure

The examination of a trap location is herein referred to as ‘sampling’, independent of the presence or absence of mosquito eggs, larvae or pupae. Dried or toppled ovitraps were excluded from further analysis, while missing traps were replaced. If the trap water contained any larvae or pupae, these were transferred to a glass jar along with the remaining trap water which, if necessary, was adjusted to 100 ml volume (positive sample). Otherwise, the trap water was discharged, and the ovitraps were refilled with fresh water (negative sample). The masonite sticks were checked for eggs in the field; when present, the eggs were taken to the laboratory and put into water for the larvae to hatch (the sample was considered positive when larvae hatched). Removed sticks were replaced. The larvae and pupae were maintained in the laboratory until the emergence of adults, which were captured and stored in vials at −20 °C. All adults and dead larvae were identified morphologically using the keys of Becker et al. [41] and Schaffner et al. [42]. For comparative analysis, the numbers of adults hatched from collected eggs, larvae and/or pupae per trap were recorded. A sample in which many eggs collapsed or all of the larvae died during transport or in the laboratory was included in the dataset as positive for the specific mosquito taxa. Twenty-two *Cx. pipiens* s.l. individuals of randomly selected samples of all studied land use types were determined genetically to species and biotype level by real-time polymerase chain reaction (PCR) targeting the ace2 and CQ11 microsatellite loci [43].

The number of ovitraps colonised by mosquito specimens related to the total number of ovitraps indicated the occurrence of the respective species {container index (CI), also known as deposit index [44]}. We followed Focks [45] in the interpretation of these data, who categorised the CI according to density, i.e. a CI of 1–9 indicates a low density, 10–27 a medium density and > 27 a high density. The distribution of land use (the proportions of arable land, forest or settlement in a 100 m-radius around the trap location) was calculated using QGIS version 3.2 (with functions buffer and union). This was also used for the figures showing the trap locations at the respective study sites. Differences between land use types and trap locations were tested using Fisher’s exact test with post hoc adjustment using the Benjamini and Yekutieli correction [46]. Data management, statistics and diagrams were done using Microsoft Excel 2010, IBM SPSS 22.0 and R Studio 1.1.383 with R version 3.6.0 [47] using the packages foreign [48], readxl [49] and rcompanion [50].

### Statistical analysis

The distribution of mosquito taxa along the environmental gradients was analysed with a canonical correspondence analysis (CCA) using R package vegan [51], applying the environmental data (proportions of arable land, forest, settlement area) as the predictors and site-specific mosquito occurrence data as the response in linear combinations. The gradient axes represent linear combinations of the environmental variables, and maximum abundance of the taxa is represented by points calculated near the corresponding axes [52].

To test the effects of several predictor variables on *Ae. japonicus japonicus* occurrence, we firstly pre-selected different models (Gaussian, Poisson, negative binomial and zero-inflated) and tested model fit by comparing Akaike information criterion (AIC) values and rootogram output [53, 54]. As predictors, information collected during field studies, such as water temperature, occurrence of native taxa, location of the trap according to land use type and number of tree species in a 10 m-radius of the trap locations was used (for the full list of variables, see Table 3).

The test for multicollinearity by calculation of variance inflation factors returned values of < 4 for all predictor variables. For the most accurate model,* R*^2^- and adjusted* R*^2^-values were calculated following Nagelkerke [55]. Logistic regression of the proportional occurrence of *Ae. japonicus japonicus* vs. the most abundant native taxa was calculated after the method of Byrd et al. [23] with quasibinomial distribution. Models and visualisations were conducted with R packages car [56], countreg [57], cowplot [58], ggplot2 [59], MuMIn [60] and pscl [61].

## Results

### Trap type preference and co-occurrence of mosquito taxa

As we did not obtain a statistically significant effect of trap type on the frequency of *Ae. japonicus japonicus*-positive ovitraps (Fisher’s exact test: two sided, *P* = 0.093), respective counts were summarised for each trap location. In 2017, we did 1000 samplings, of which 372 were excluded from further analysis as the traps contained no water, mostly due to high temperatures and desiccation during the summer. As the first examination revealed no mosquito-positive ovitraps, all samplings from this date were excluded from further analysis, resulting in valid samplings from five examination dates. We discovered eggs, larvae and pupae of four different mosquito taxa in 381 ovitraps, resulting in 60.7% mosquito-positive traps of all analysable traps. *Ae. japonicus japonicus* was found in 85 and 12 samplings in Alfter and Dormagen, respectively, totalling 15.4% of all analysable samplings (Table 1). *Cx. pipiens* s.l. was detected in 199 samplings, corresponding to 31.7% of all samples, and significantly more frequently in plastic cups (36% traps positive) than in wood blocks (25% traps positive) (Fisher’s exact test: *P* = 0.004). Furthermore, 47 ovitraps contained *An. plumbeus* (7.5% of all samples) and 38 *Ae. geniculatus* (6.1% of all samples). Eighty ovitraps (21.0% of all analysable traps) were colonised by more than one species (Table 1). In most cases (*n* = 24), *Cx. pipiens* s.l. and *Ae. japonicus japonicus* were found in the same trap, followed by the combinations *Cx. pipiens* s.l. and *An. plumbeus* (*n* = 12), and *Ae. japonicus japonicus* and *An. plumbeus* (*n* = 10) (Additional file 1: Table S1).

**Table 1:** Total number and percentages of positive samples and occurrence of mosquito species per trap

Study		*Aedes japonicus japonicus*	*Culex pipiens* s.l.	*Anopheles plumbeus*	*Aedes geniculatus*	Total
2017	Total positive traps (*n*)	97	199	47	38	381
	Positive traps/analysable traps (%)	15.4	31.7	7.5	6.1	60.7
	Traps multiple species (*n*)	56	58	39	25	80
	Multiple species/positive traps (%)	57.7	29.1	83	65.8	21
2018	Total positive traps (*n*)	441	285	137	19	882
	Positive traps/analysable traps (%)	20.3	13.1	6.3	0.9	40.7
	Traps multiple species (*n*)	180	139	113	11	206
	Multiple species/positive traps (%)	40.8	48.8	82.5	57.9	23.4

Calculations based on a total of 628 samples in 2017 and 2168 samples in 2018. The number of positive ovitraps with more than one species divided by the total number of positive ovitraps represents the portion of positive ovitraps with multiple species. See Additional file 1: Table S1 for all combinations of species and Additional file 2: dataset S1 for all samplings

In the 2018 field study, 532 of the 2700 samplings were discarded from the analysis because the ovitraps dried up, were knocked over by animals or removed by humans. *Ae. japonicus japonicus* was the most frequently trapped mosquito taxon and found in 441 (20.3%), *Cx. pipiens* s.l. in 284 (13.1%), *An. plumbeus* in 137 (6.3%) and *Ae. geniculatus* in 19 (0.9%) out of 2168 samples (Table 1). Of 882 positive ovitraps (40.7% of all analysable traps), 175 contained two and 31 three different mosquito taxa (23.4% of all analysable traps). Syntopic occurrence was observed most frequently for *An. plumbeus* (83.0 and 82.5% in 2017 and 2018, respectively), followed by *Ae. geniculatus* (65.8 and 57.9%), *Ae. japonicus japonicus* (57.7 and 40.8%) and *Cx. pipiens* s.l. (29.1 and 48.8%) as additional species (Table 1). In most cases (*n* = 84), *Cx. pipiens* s.l. and *Ae. japonicus japonicus* were found in the same trap, followed by the combinations *Ae. japonicus japonicus* and *An. plumbeus* (*n* = 58), and *Cx. pipiens* s.l. and *An. plumbeus* (*n* = 24) (Additional file 1: Table S1). Habitat sharing was most common in the forest–settlement transect, with the combination *Cx. pipiens* s.l. and *Ae. japonicus japonicus* [*n* = 19 at the forest trap location 10 m from the transition zone (F10) and at the settlement trap location 10 m from the transition zone (S10), respectively] and the combination *Ae. japonicus japonicus* and *An. plumbeus* [*n* = 18 at trap location F10 and *n* = 20 at the forest trap location 100 m from the transition zone (F100)] (Additional file 1: Table S1).

### Effects of air temperature on oviposition

**Fig. 3:**
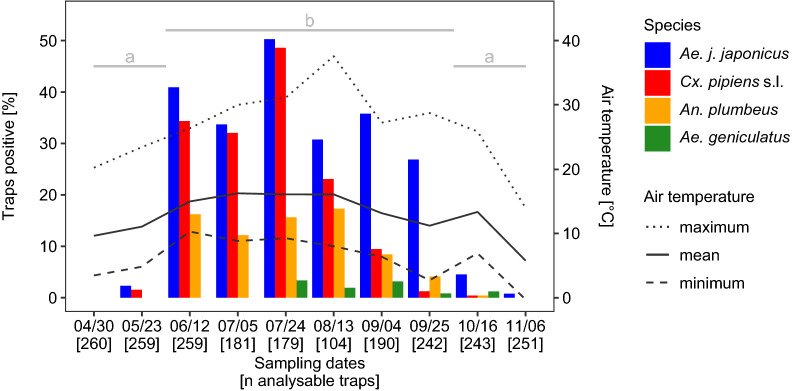
Percentages of mosquito-positive ovitraps (*Traps positive*), and air temperatures. Air temperature was calculated in the week before sampling in 2018. Sampling dates and the number of analysable ovitraps from a total of 270 ovitraps (*in brackets*) are shown on the x-axis. There was no statistically significant difference between the numbers of *Aedes japonicus japonicus*-positive ovitraps sampled in spring (April–May) and in autumn (October–November) (*identical lowercase letters*). By contrast, the numbers of *Ae. japonicus japonicus*-positive ovitraps in the summer (12 June to 25 September) were statistically significantly higher than in spring and autumn (*different lowercase letters*) (Fisher’s exact test: *P* < 0.0001. For all *P*-values, see Additional file 1: Table S2. For total numbers of emerged adults, see Additional file 2: dataset S1

To analyse the seasonal occurrence of the mosquito taxa, we used mosquito collection data from April to November 2018, as the field study in that year started earlier and ended later than the 2017 field study. A warm January with an above-average mean temperature (3.8 °C) in North Rhine-Westphalia with large frost-free regions in the study area was followed by an unusually sunny and dry February with a mean temperature of −0.9 °C [62]. The mean temperature in March was 3.8 °C; there were two periods of frost in March, at the beginning and in the middle of the month [62]. With the warmest April since records started in 1881 (mean of 12.8 °C in North Rhine-Westphalia) [62]), spring and summer began almost simultaneously. May was also warmer than usual (compared to the mean of the international reference period from 1961 to 1990), and some thunderstorms provided the region with plenty of rain [62]. The summer (June–September) was characterised by low precipitation (115 l/m^2^; cf. 240 l/m^2^ for 1961–1990), a lot of sunshine (740 h; cf. 554 h for 1961–1990) and a mean temperature of 19.3 °C (cf. 16.3 °C for 1961–1990) [62]. This trend continued into the beginning of October. By the end of October the temperatures dropped considerably and reached below zero between 16 October and 6 November (authors’ measurements; Fig. 3).

After two inspections in April and May where no or very few mosquito eggs, larvae or pupae were recorded, a considerable increase in the number of positive traps was recorded in early summer, which peaked at the end of July (Fig. 3). The maximum air temperature, 36.6 °C, was never measured for more than 1 h at *Ae. japonicus japonicus*-positive trap locations. In spite of air temperatures of ca. 40 °C, water temperatures of up to 30 °C, and 60% of the ovitraps drying up, many of the ovitraps were colonised by all four mosquito taxa in August, before the proportion of native mosquitoes declined till late September, in contrast to *Ae. japonicus japonicus*. When the mean temperature dropped below 14 °C, *Ae. japonicus japonicus* numbers also decreased, until only two ovitraps were found to be occupied by this subspecies during the last trap inspection.

The seasonal occurrence of *Ae. japonicus japonicus* eggs, larvae or pupae showed two major, statistically significantly different groups (Fisher’s exact test: *P* < 0.0001): spring (April/May) and autumn (October/November) [low numbers of positive ovitraps (< 5%)]; and summer (from 12 June until 25 September) [high numbers of positive ovitraps (> 25%)]. *P-*values of comparisons for sampling dates which indicated no significant differences are included in Additional file 1: Table S2.

### Effects of land use type on oviposition: 2017 field study

The CI of the forest–settlement transect showed the highest number of ovitraps positive for *Ae. japonicus japonicus* at both study sites (43% in Alfter, 10% in Dormagen) (Additional file 1: Table S3). The transition zone in this transect featured the most *Ae. japonicus japonicus-*positive ovitraps, with 66% in Alfter and 28% in Dormagen (Additional file 1: Table S3). Significantly fewer *Ae. japonicus japonicus*-positive ovitraps were detected along the arable land–forest (Fisher’s exact test: *P* = 0.0045; 27% in Alfter, 3% in Dormagen), and arable land–settlement transects (Fisher’s exact test: *P* < 0.0001; 3% in Alfter, 2% in Dormagen) and none along the settlement–settlement transect.

The most frequent mosquito taxon in the ovitraps, *Cx. pipiens* s.l., was found at almost every location (mean CI 15–50%), with no significant differences between trap locations related to transect types (Fisher’s exact test: *P* = 1.00 for arable land–forest, arable land–settlement and forest–settlement). The real-time PCR of selected *Cx. pipiens* s.l. samples indicated seven *Culex pipiens* biotype *pipiens* Linnaeus 1758 and 15 *Culex torrentium* Martini 1925, with the first originating from traps in arable land, forest or transition zones between forest and arable land or forest and settlements. The *Cx. torrentium* samples were from trap locations in all studied land use types, including the centre of the settlement areas. Both taxa occurred in samples taken in June, July and August.

**Fig. 4:**
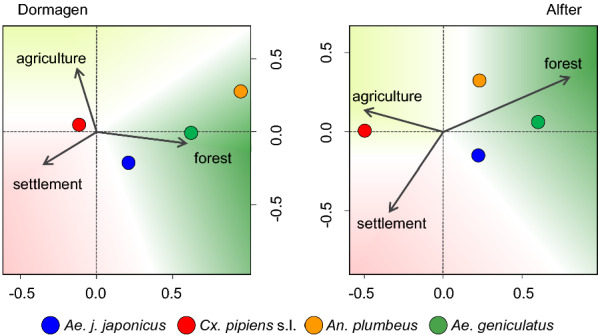
Canonical correspondence analysis of land use types and mosquito taxa. *Ae. j*. *japonicus* occupied significantly more positive ovitraps in the settlement–forest transition zone than in other trap locations (Fisher’s exact test: *P* = 0.0045 tested against arable land–forest, *P* < 0.0001 tested against arable land–settlement)

The transition zone in the forest–settlement transect and a trap site 10 m into the forest exhibited the highest numbers of *An. plumbeus*-positive ovitraps. Similarly high numbers were found along the arable land–forest transect at the trap locations 100 m into the forest and 10 m into the forest. *Ae. geniculatus* constituted the least frequent mosquito species in the ovitraps at the study sites. It occurred mainly at trap locations in the forest (Additional file 1: Table S3). Despite the large-scale landscape differences, the mosquito-positive ovitraps were similarly distributed on a smaller scale between the study sites (i.e. on the specific transects), according to the CCA (Fig. 4). In summary, *Ae. japonicus japonicus* mainly occurred in the forest–settlement transition zone, *An. plumbeus* and *Ae. geniculatus* in the forest and *Cx. pipiens* s.l. closer to arable land and the settlement area.

### Effects of land use type on oviposition: 2018 field study

**Fig. 5:**
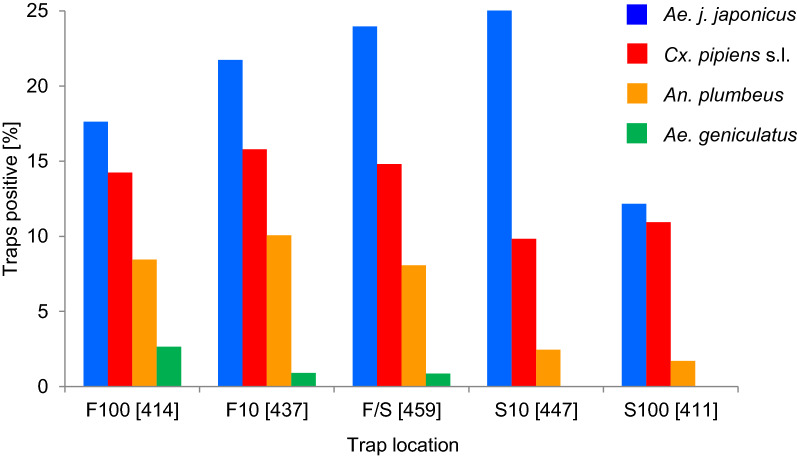
Occurrence of mosquito taxa in relation to trap location. The number of analysable traps is given *in brackets*. For abbreviations, see Fig. 2

*Ae. japonicus japonicus*-positive ovitraps occurred significantly more frequently (Fisher’s exact test: *P* = 0.0021) in the forest–settlement transition zone than in the settlement area, irrespective of forest type (Fig. 5).

We found 10–15% ovitraps positive for *Cx. pipiens* s.l., with no significant differences between trap locations [Fisher’s exact test: *P* = 1.00 for F100 vs. F10, F100 vs. F/S, F10 vs F/S, F100 vs. settlement trap location 10 m from the transition zone (S100), S10 vs. S100; *P* = 0.78 for F/S vs. S100; *P* = 0.53 for F100 vs. S10; *P* = 0.29 for F10 vs. S100; *P* = 0.23 for F/S vs. S10; *P* = 0.07 for F10 vs. S10]. The ovitraps positive for *An. plumbeus* and *Ae. geniculatus* were mainly located in the forest, with significantly fewer ovitraps positive in the settlement area (there were only sufficient numbers of samples for statistical analysis for *An. plumbeus*; Fisher’s exact test: *P* = 0.0009 for S10 and S100 compared to F100, F10 and F/S). According to heatmaps showing the seasonal variance of the land use-dependent occurrence of the mosquito taxa (Additional file 1: Fig. S3), the first *Ae. j*. *japonicus*-positive ovitraps were found in S10 and in the forest, *Cx. pipiens* s.l. was recorded first in the settlement area, and *An. plumbeus* in the forest and the transition zone. From 24 July to 4 September, the three taxa occurred in all land use types. In autumn, *Ae. j*. *japonicus* appeared mainly in the transition zone, whereas the last few ovitraps positive for *Cx. pipiens* s.l. were found in the settlement area and those positive for *An. plumbeus* primarily in the forest. The occurrence of mosquito taxa differed only slightly between trap locations in different forest types, and no taxon showed a significant preference (Fisher’s exact test: *P* = 0.72 for *Ae. japonicus japonicus* in deciduous vs. coniferous, *P* = 0.41 for coniferous vs. mixed, *P* = 0.16 for mixed vs. deciduous forest; *P* = 1.00 for *Cx. pipiens* s.l. irrespective of forest type; *P* = 1.00 for *An. plumbeus* in deciduous vs. coniferous and mixed vs. deciduous forest, *P* = 0.39 for coniferous vs. mixed forest).

High numbers of positive ovitraps were observed for *Ae. japonicus* *japonicus* during the summer along the forest–settlement transect, with very high CIs in the transition zone (Additional file 1: Fig. S3). Medium numbers were found at the end of September 2018 and along the forest–arable land transect. Analysis of ovitraps sampled in May, October and November and along the arable land–settlement and settlement–settlement transects showed low CIs (Additional file 1: Table S3 and Fig. S3).

### Effects of water temperature on oviposition

**Fig. 6:**
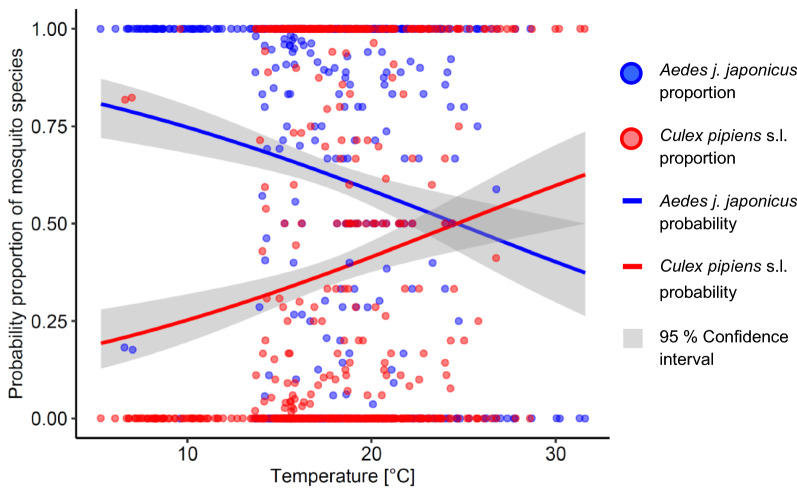
Logistic regression of probability proportion of adults hatched from larvae of *Cx. pipiens* s.l. vs*. Ae. japonicus japonicus* collected at the specified temperatures. Data from 2017 and 2018 (23 May to 25 September) for ovitraps located along the forest–settlement transect. Function jitter was used for taxa proportion data to improve visibility [85]

The maximum water temperature of *Ae. japonicus japonicus-*positive ovitraps amongst all measurements (2017–2018) was 28.6 °C on 24 July 2018 (Table 2). *Cx. pipiens* s.l. occurred more frequently in ovitraps with higher water temperature compared to *Ae. japonicus japonicus* and vice versa. The lowest observed water temperature in *Ae. japonicus japonicus*-positive ovitraps was 5.3 °C on 25 September 2018. In total, 39 *Ae. japonicus japonicus*-positive ovitraps were recorded with a water temperature < 10 °C. According to the 2017 data of the forest–settlement transect and 2018 data collected from 23 May to 25 September, the probability of oviposition by *Cx. pipiens* s.l. increased with rising temperatures, and the odds of collecting *Ae. japonicus japonicus* decreased by 0.48/1 °C (*t*-test, *t*_(683)_ = − 3.98, *P* < 0.0001). At 25 °C, the regression lines of the two taxa cross, with a probability proportion of 0.5 (Fig. 6).

**Table 2:** Maximum temperature of water (°C) in the mosquito-positive ovitraps during the field studies at the different study sites

Study	Site	*Ae. japonicus japonicus*	*Cx. pipiens* s.l.	*An. plumbeus*	*Ae. geniculatus*
2017	Alfter	26.8	34.7	31.3	20.3
	Dormagen	22.1	27.2	17.3	21.7
2018	Alfter	22.6	19.8	20.6	n.n.
	Bonn Süd	24.7	24	17.3	n.n.
	Heimerzheim	24.3	24.6	23.2	22.7
	Lohmar	28.6	25.8	28.6	16.6
	Siegburg	25.8	30	27	23.1
	Troisdorf	26.8	27.7	25.2	n.n.

*n.n.* Species not present

### Effects of water temperature, tree species, trap location and native mosquitoes on oviposition of *Ae. japonicus japonicus*

The negative binomial model returned the lowest AIC results relative to the other methods (for AIC-values and rootograms see Additional file 1: Fig. S4) and was used further. Four significant predictors (*R*^2^ = 0.2773, adjusted* R*^2^ = 0.2785) were positively associated with *Ae. japonicus japonicus* counts: mean temperature, use of the same microhabitat as an oviposition site by *An. plumbeus*, and two trap locations—one in a transition zone (F/S) and one 10 m from the transition zone towards a settlement (10S) (Table 3). Hence, with increasing mean water temperature the expected count of ovitraps positive for *Ae. japonicus japonicus* increased (by e^0.2126^ = 1.2369) and the use of a trap location for oviposition by *An. plumbeus* multiplied the expected count (by the factor e^0.5999^ = 1.8219). Similarly, positive effects were caused by trap location at or near a transition zone, 10 m into the settlement area.

**Table 3:** Coefficients and statistically significant output of predictor variables as calculated by the generalised linear model

	Estimate	SE	*Z*-value	*P*
(Intercept)	− 29.890	15.100	− 19.790	0.0478*
Temp_mean	0.2126	0.0933	22.800	0.0226*
Cxbin	0.2732	0.3148	0.868	0.3854
Plbbin	0.5999	0.2253	26.630	0.0077*
F100	0.4931	0.4826	10.220	0.3068
F10	0.7486	0.4127	18,140	0.0697
F/S	0.8715	0.3728	23.380	0.0194*
S10	10.770	0.3347	32.170	0.0013*
Ngbi	0.0412	0.1157	0.356	0.722
Nhbu	0.0001	0.0251	0.003	0.9972
Ngki	0.0295	0.0432	0.684	0.4941
Nsei	− 0.0362	0.0609	− 0.594	0.5528
Ntei	− 0.0845	0.0917	− 0.922	0.3566
Ngfi	0.1002	0.0832	12.050	0.2283
Nrbu	0.0648	0.0456	14.200	0.1557

Characteristics: negative binomial, link = log,* z*-values calculated by Wald-test. Response variable: total of *Ae. japonicus japonicus*-positive ovitraps per location

Predictors: Temp_mean = Mean water temperature, binary native taxa occurrence: Cxbin = *Cx. pipiens* s.l.,* Plbbin* *An. plumbeus*, land use data: percentage forest: F100 = 100% forest, F10 = 60% forest, F/S = 50% forest, S10 = 40% forest, number of tree species in a 10 m radius of the trap locations (the tree species occurred in more than five transects): Nrbu: *Fagus sylvatica*, Nhbu: *Carpinus betulus*, Ngbi: *Betula pendula*, Nsei: *Quercus robur*, Ntei: *Quercus petreae*, Ngfi: *Picea abies*, Ngki: *Pinus sylvestris*

* *P* < 0.05

 The numbers of the most abundant tree species had no significant effect on the frequency of ovitraps containing *Ae. japonicus japonicus* (Table 3). Thus, specific tree species did not have an effect on the oviposition of the Asian bush mosquito in this study.

## Discussion

The taxa that most frequently colonised the traps in this study were *Cx. pipiens* s.l. and *Ae. japonicus japonicus*, followed by *An. plumbeus* and *Ae. geniculatus* (Table 1). Whereas *Cx. pipiens* s.l. occurred mainly in the summer without showing a preference for land use-related oviposition, *An. plumbeus* and *Ae. geniculatus* were found almost exclusively at trap locations in the forest (Fig. 5). When the numbers of native mosquitoes dropped at the end of September, up to 30% of all traps were still found to be *Ae. japonicus japonicus*-positive (Fig. 3). This invasive subspecies occurred more frequently at rural compared to suburban sites and was more prevalent in the transition zone between forest and settlement areas compared to trap locations in other land use types (Additional file 1: Table S3). *An. plumbeus* shared an oviposition and larval microhabitat more often with *Ae. japonicus japonicus* than with the other native species (Table 3; Additional file 1: Table S1). Temperatures higher than 30 °C and the arable land and settlement land use types seemed to have a negative effect on the occurrence of *Ae. japonicus japonicus* (Fig. 6; Additional file 1: Table S3)*.*

*Ae. japonicus japonicus* has spread across large regions of Germany in the 11 years since its first known occurrence in the country [13, 16, 63]. This subspecies is regularly reported during active or passive monitoring in densely populated regions like North Rhine-Westphalia [13, 64]. This is reflected in our transect data for 2018, where *Ae. japonicus japonicus* occurred at all the study sites at least once during the sampling period. However, our results indicate large differences in the occurrence of the Asian bush mosquito on a regional and local scale, depending on the degree of rurality or suburban character of a region, and especially regarding the mosquito’s preference for, and avoidance of, specific land use types.

### Phenology

The low numbers of mosquito-positive traps in spring (Fig. 3; Additional file 2: dataset S1) were probably due to two late frost events in March, during which emerging adults from overwintering larvae or larvae hatched from overwintering eggs of *Ae. japonicus japonicus* could have been killed. The month of April was very warm but too dry to induce egg hatching. Only in May did thunderstorms with intense rainfall occur, which presumably refilled natural and artificial oviposition habitats sufficiently to enable comprehensive hatching of overwintering eggs.

The maximum number of traps positive for mosquito taxa ovipositing in containers were counted in summer, at the end of July 2018. *Ae. japonicus japonicus* was found in 50% of all samples and remained the most common mosquito taxon at the study sites until the end of September. By that time, native taxa were much less common, presumably due to the decreasing temperatures in autumn. Our research corroborates previous findings [9, 26, 65] that *Ae. japonicus japonicus* is well adapted to temperate climates (temperate oceanic and humid continental climate after improved Köppen-Geiger classification [66]) and better able to develop at lower temperatures than many native taxa [34, 35]. This adaption gives this newcomer an advantage over competing indigenous mosquito taxa. The numbers of *Cx. pipiens* s.l.-positive traps decreased with falling temperatures in the end of summer (Fig. 3), which was also observed in a study from Italy [67].

### Land use

According to the results of the present study, *Ae. japonicus japonicus* appears to prefer the transition zone between forest and settlement area as an oviposition habitat (Figs. 4, 5). Traps in the centre of settlement areas, 500 m away from natural oviposition sites, as well as at locations on arable land and settlement areas at a distance of 100 m from the forest were never or only occasionally colonised (Additional file 1: Table S3). Far fewer ovitraps were occupied by *Ae. japonicus japonicus* at the rather suburban site of Dormagen compared to the more rural site of Alfter (Additional file 1: Table S3). Ovitraps in mixed forests seemed to be more frequently colonised, but the differences were not significant. The first *Ae. japonicus japonicus*-positive traps identified in May were in locations in land use types S10 and F100, and were presumably due to oviposition by females that had developed from eggs overwintering in vases, rain barrels or tree holes. With increasing temperatures, the number of positive traps increased in all land use types, with a maximum at the end of July in the transition zone. Based on the decreasing numbers of positive traps, we assume that the temperatures in August were above optimum for this subspecies at some trap locations (Fig. 3; Additional file 1: Fig. S3). In September and October, most traps were colonised in the transition zone, continuing the trend from July.

These data correlate well with previous studies. Results from Japan indicated oviposition mainly in rural and more densely vegetated areas [68], but also in ovitraps on concrete on the ground close to forests consisting mainly of bamboo and oak [24]. In a study carried out in the USA, higher numbers of eggs were deposited in ovitraps at the forest edge compared to the forest interior [28], and higher abundances of pupae occurred in rural compared to urban and suburban areas [26]. In a recent study from Hungary, forest patches (smaller than 500 ha) and vineyards were positively correlated with the occurrence of *Ae. japonicus japonicus*, and it was assumed that these areas represent corridors and larger continuous forests and arable land barriers for the spread of the subspecies [32]. This conjecture, together with the results from our study, could be an explanation for the slow spread of the subspecies in the federal state of Lower Saxony [65], whose landscape is mostly shaped by agriculture, and its absence in the federal state of Brandenburg [13], whose landscape is dominated by large coniferous forests.

Spatial gradients in transition zones influence biotic and abiotic conditions such as microclimate (e.g. forest interiors are cooler than forest edges, and winds are weaker and less turbulent) or biotic factors (e.g. more leaf litter is found in forest interiors compared to forest edges) [69]. Some of these indirect effects of different land use types may be the main reasons for the oviposition preferences of mosquitoes. Hot areas in arable land and settlements could act as a temperature barrier for the distribution of *Ae. japonicus japonicus*, as the subspecies was only rarely found in the present study in these types of land use. The increased number of positive traps in the forest and in transition zones compared to arable land and settlement area suggests that *Ae. japonicus japonicus* is a forest-related taxon. However, oviposition at places penetrating 10 m into apparently unsuitable areas (arable land, settlement area) indicates the readiness of *Ae. japonicus japonicus* to use artificial oviposition sites that are more distant from the forest, which could give the introduced subspecies an advantage over more conservative native forest species like *An. plumbeus* and *Ae. geniculatus*.

In the present study, *Ae. japonicus japonicus* showed no preference for artificial (plastic cups) or natural (logs of beech) trap types, but *Cx. pipiens* s.l. was found significantly more frequently in artificial traps. *Cx. torrentium* was found at trap locations in all land use types. This could be due to the eurytopic character [33] or the strong dispersal capacity of this species [70]. Based on the genetic differentiation of *Cx. pipiens* samples, a preference of *Cx. pipiens* biotype *pipiens* can be assumed for ovitrap locations in (semi)natural environments*.* In Portugal, *Cx. pipiens* biotype *pipiens* was observed feeding in shelters used for animals and sylvan habitats [71], whereas *Cx. pipiens* biotype *molestus* Forskal, 1775 shows more stenogamous and anthropophilic behaviour [72]. As only a small sample of *Cx. pipiens* s.l. was tested for species and biotype, further research regarding the land use preferences of the biotypes of this species complex should clarify if the above assumption points in the right direction.

The presence of specific tree species had no effect on the number of *Ae. japonicus japonicus*-positive ovitraps. The effects of leaf litter on mosquito larvae depend on the tree species, and can be beneficial (e.g. when quickly decomposing leaves are available) or detrimental (e.g. the presence of plant tannins or other phenolics to which some mosquito species are sensitive) [73, 74]. We hypothesise that taxa like *Ae. japonicus japonicus* and *An. plumbeus*, whose larvae are regularly found in tree holes, are relatively tolerant to many secondary plant compounds (tannins etc.), which would explain that there was no observable effect of tree species in the present study.

The use of CI to estimate yellow fever risk [44] has been criticised because it does not consider the size and, thus, the different productivity potential of containers [45]. Despite this, ecological studies use CI as a measure of mosquito abundance patterns, preference of oviposition conditions (shady vs. sunlit areas) or preference of container material (e.g. [75]). The fact that the highest CIs for *Ae. japonicus japonicus* were during the summer and along the forest–settlement transect underpins the preference of this subspecies for this season and land use type. Based on these findings, further studies should incorporate sampling of available oviposition sites (e.g. rain water barrels, discarded tyres, flower vases) to give a more accurate estimation of oviposition habitat preference.

### Temperature and co-occurrence

In the present study, larvae of *Ae. japonicus japonicus* were never found in traps with water temperatures higher than 29 °C. The maximum constant water temperature for the development of larvae of a North American *Ae. japonicus japonicus* strain in the laboratory was 28 °C, and larvae survived until the third instar at 34 °C [20]. In a recent laboratory study from Germany, the mean mortality rate of larvae at a constant maximum of 31 °C was 87.5% [21].

The logistic regression analysis of the water temperature-dependent proportion of *Ae. japonicus japonicus* vs. *Cx. pipiens* s.l. revealed a preference of the introduced subspecies for colder temperatures, in contrast to warmer water temperatures with significantly higher proportions of the native taxon (Fig. 6). Depending on the oviposition and larval habitat, this can result in an advantage for *Ae. japonicus japonicus* (in the transition zone between forest and settlement area) at water temperatures up to 25 °C, whereas *Cx. pipiens* s.l. (in this case presumably *Cx. torrentium*) is apparently well adapted to other land use types unaffected by temperature, as demonstrated by its corresponding higher proportions. Independent of climate change scenarios, and unlike *Ae. japonicus japonicus*, *Cx. pipiens* s.l. generally benefits from warm water temperatures in natural or artificial containers. Depending on the container type (e.g. tree hole at the base of a trunk), the water temperature may be much lower than the air temperature, resulting in a microhabitat offering shelter for eggs and/or larvae from extreme temperatures [76]. Utilisation of such microhabitats may help *Ae. japonicus japonicus* to survive hot summers, like those in 2018 and 2019.

So far, studies on competition between *Ae. japonicus japonicus* and native mosquito species have not been conducted in Germany, but a slight advantage of *Cx. pipiens* biotype *molestus* over *Aedes albopictus* (Skuse, 1895) was detected in one study [77]. In the present study, co-occurrence was observed for all four mosquito taxa (Table 1), and most frequently for *An. plumbeus*. The results from the generalised linear model (Table 3) confirmed that *An. plumbeus* and *Ae. japonicus japonicus* use similar types of microhabitats as oviposition sites. In the laboratory, we observed that larvae of *Ae. japonicus japonicus* usually hatched quickly when the eggs were submerged in water; even contact with moist paper tissue induced eclosion. In contrast, larvae of *An. plumbeus* were noticed mostly after the pupation of *Cx. pipiens* s.l. and *Ae. japonicus japonicus.* During and after the emergence of adult mosquitoes the water became cloudy, probably due to increased microbial activity resulting in a decreasing amount of dissolved oxygen, a known hatching stimulus for many *Aedes* species ovipositing in artificial and natural containers [78]. In contrast, the invasive species *Aedes aegypti* (Linnaeus, 1762),* Ae. albopictus* and *Ae. japonicus japonicus* are able to eclose immediately after contact with tap water or deionised water [21, 79, 80], although the hatching rate of the two former species is much higher in nutrient broth [81]. Their ability to eclose at normal concentrations of oxygen gives them temporal advantage over many native species, in this case *An. plumbeus* and *Ae. geniculatus*.

*Ae. japonicus japonicus* is able to coexist with other mosquito species in many larval habitats. In our study, this invasive subspecies was found frequently in traps with *An. plumbeus* at trap locations in the forest and with *Cx. pipiens* s.l. in all land use types except for the arable land–settlement transect (Additional file 1: Table S1). Co-occurrence of larvae of the Asian bush mosquito was also reported for *Culex hortensis* Ficalbi, 1889,* Cx. pipiens* s.l.,* Ae. albopictus, Culiseta longiareolata* (Macquart, 1838), *Ae. geniculatus* and *Culiseta annulata* in artificial microhabitats [82]. The occurrence of *Ae. japonicus japonicus* seems, separate from its land use preferences, only limited by higher water temperatures (Table 2), which can give an advantage to heat-tolerant larvae of native mosquito species (Fig. 6). A similar observation was made in the USA, where *Ae. atropalpus* colonised rock pools with temperatures of up to 39.8 °C, in contrast to *Ae. japonicus japonicus*, which was significantly more frequent at temperatures of less than 30 °C [23].

In the present investigation we trapped native species (Table 1) which are potential vectors of disease agents [83]. Individuals of the ornithophilic *Cx. pipiens* biotype *pipiens* as well as hybrids of this taxon and the more anthropophilic *Cx. pipiens* biotype *molestus* were recently found to be WNV positive in a wildlife park in Germany [84]. Although *Ae. japonicus japonicus* has been shown to be an opportunistic feeder in human environments [6] and vector competent for various pathogens [83], this subspecies is not more or less dangerous than native species. Yet, it adds to the risk of becoming infected with a pathogen transmitted by a blood-feeding insect in Germany.

## Conclusions

The present study revealed the period of main oviposition activity (June–September), the tolerated water temperature range (5–29 °C) and favoured oviposition habitat (forest–settlement transition zone) of the Asian bush mosquito *Ae. japonicus japonicus*. According to the results of this study, the occurrence of this subspecies is not associated with specific tree species, which suggests that it has a broad tolerance of secondary plant compounds; this may facilitate its spread into areas with plants that do not occur in its area of origin. As the study area lacked large coniferous forests, it is recommended that appropriate areas should be examined for the presence of *Ae. japonicus japonicus* in Baden-Wuerttemberg or Bavaria, where coniferous forests, dominated by European spruce *Picea abies*, are larger and more common*.* In contrast to container-inhabiting native mosquito taxa, *Ae. japonicus japonicus* was demonstrated to colonise most of the traps in its preferred oviposition habitat in western Germany. Against the background of it being an opportunistic feeder, this underpins its importance as a relevant potential vector of WNV. Co-occurring native mosquito taxa have shorter seasonal oviposition activity, but are able to tolerate higher temperatures (*Cx. pipiens* s.l.). The frequently co-occurring native species *An. plumbeus* could be used as an indicator for potentially suitable oviposition sites for *Ae. japonicus japonicus* in hitherto uncolonised regions. There is no indication that native mosquito species are displaced by *Ae. japonicus japonicus.* However, this should be studied in natural oviposition sites like tree holes and rock pools where resources are limited in comparison to large artificial containers like rain water barrels or regularly refilled vases in cemeteries. The results reported here for *Ae. japonicus japonicus* are in accordance with those of studies from other countries, and also confirm some assumptions that have been made for modelling approaches, e.g. that arable land and water temperatures of more than 30 °C represent distribution barriers [38]. It can be concluded that this subspecies appears to spread and establish where settlements meet areas with small patches of forest, which is the situation in most parts of southern and western Germany. In contrast, less densely populated regions with areas of land dominated by agriculture or large forests, which is the situation in large regions of northern and eastern Germany, seem to represent distribution barriers to this subspecies. At the present time, this conclusion is supported by the known distribution area of the Asian bush mosquito in Germany [13]. The new insights presented here related to the land use-dependent occurrence of mosquitoes, especially regarding transition zones, and the consideration of native species, contribute to a better understanding of mosquito ecology and provide data for more targeted monitoring, distribution modelling and risk management of these species.

## Supplementary information

**Additional file 1: Fig. S1.** Study site 2017 at Dormagen with trap locations as related to land use type. **Fig. S2**. Study site 2017 at Alfter with trap locations as related to land use type. Due to logistical reasons, two transects overlap. **Fig. S3**. Heatmaps showing occurrence of mosquito taxa [*Aedes japonicus japonicus* (*a*), *Culex pipiens* s.l. (*b*), *Anopheles plumbeus* (*c*); mean positive ovitraps per trap location] in relation to trap location.* F100* Forest, 100 m from the transition zone;* F10* forest, 10 m from the transition zone;* F/S* transition zone;* S10* settlement, 10 m from the transition zone;* S100* settlement, 100 m from the transition zone. For the number of analysable samplings, see Fig. 3. **Fig. S4.** Rootograms of the model with different distribution types. Akaike information criterion (*AIC*) values: Gaussian = 498.9, Poisson = 538.4, zero-inflated = 483.2, negative binomial = 467.5. **Table S1.** List of traps positive for species and species combinations at trap locations in different land use types.* C* *Cx. pipiens* s.l.,* J* *Ae. japonicus japonicus*,* G* *Ae. geniculatus*,* P* *An. plumbeus*,* A* arable land,* S* settlement area,* F* forest.* Numbers in the description of the trap location* indicate distance (m) to the transition zone, which is marked by a* diagonal slash* (e.g.* A/F* indicates arable land–forest transition zone).* Species combinations* represent numbers of traps with the specified taxa in the same trap. **Table S2**. Output of Fisher’s exact test (*P*-values) for the comparison of sampling dates by numbers of traps positive for *Ae. japonicus japonicus*. * *P* < 0.0001 **Table S3.** Land use-related oviposition in traps in Alfter and Dormagen 2017. For abbreviations, see Table S1. The mean of the portion of positive traps was tested between transects (*different uppercase letters* indicate significant difference) and trap locations (*different lowercase letters* indicate significant difference) according to Fisher’s exact test

**Additional file 2: dataset S1.** Number of adults developed from eggs, larvae or pupae, sampled at a specific date, site, and in a specific land use and trap type.* Date* Sampling date;* Site* study site;* Transect* transect type;* Land_use_type* abbreviations of land use types (see Additional file 1, Table S2);* Forest_type* abbreviation of forest type [coniferous (*C*), deciduous (*D*), mixed (*M*)];* Latitude + Longitude* coordinates of trap locations;* Trap_type* trap material (wood or plastic);* Trap_nr* consecutive trap number;* Temp* water temperature during sampling (°C);* C* *Culex pipiens* s.l.;* J* *Aedes japonicus japonicus*;* G* *Aedes geniculatus*;* P* *Anopheles plumbeus*;* Loss* cause of missing sample (trap dry, missing or toppled)

## Data Availability

All data generated or analysed during this study are included in this article (and in its additional files).

## References

[1] Sardelis MR, Turell MJ, Andre RG (2002). Laboratory transmission of La Crosse virus by *Ochlerotatus j. japonicus* (Diptera: Culicidae). J Med Entomol..

[2] Takashima I, Rosen L (1989). Horizontal and vertical transmission of Japanese encephalitis virus by *Aedes japonicus* (Diptera: Culicidae). J Med Entomol..

[3] Veronesi E, Paslaru A, Silaghi C, Tobler K, Glavinic U, Torgerson P (2018). Experimental evaluation of infection, dissemination, and transmission rates for two West Nile virus strains in European *Aedes japonicus* under a fluctuating temperature regime. Parasitol Res..

[4] Schaffner F, Vazeille M, Kaufmann C, Failloux AB, Mathis A (2011). Vector competence of *Aedes japonicus* for chikungunya and dengue viruses. Eur Mosq Bull..

[5] Abbo SR, Visser TM, Wang H, Göertz GP, Fros JJ, Abma-Henkens MHC (2020). The invasive Asian bush mosquito *Aedes japonicus* found in the Netherlands can experimentally transmit Zika virus and Usutu virus. PLoS Negl Trop Dis..

[6] Schönenberger AC, Wagner S, Tuten HC, Schaffner F, Torgerson P, Furrer S (2016). Host preferences in host-seeking and blood-fed mosquitoes in Switzerland. Med Vet Entomol..

[7] Kampen H, Kuhlisch C, Fröhlich A, Scheuch DE, Walther D (2016). Occurrence and spread of the invasive Asian bush mosquito *Aedes japonicus japonicus* (Diptera: Culicidae) in West and North Germany since detection in 2012 and 2013, respectively. PLoS ONE.

[8] Kalan K, Ivović V, Glasnović P, Buzan E (2017). Presence and potential distribution of *Aedes albopictus* and *Aedes japonicus japonicus* (Diptera: Culicidae) in Slovenia. J Med Entomol..

[9] Tanaka K, Mizusawa K, Saugstad ES (1979). A revision of the adult and larval mosquitoes of Japan (including the Ryukyu Archipelago and the Ogasawara Islands) and Korea (Diptera: Culicidae). Contrib Am Entomol Inst..

[10] Gutsevich AV, Dubitskij AM (1987). New species of mosquitoes in the fauna of the USSR. Mosq Syst..

[11] Cameron EC, Wilkerson RC, Mogi M, Miyagi I, Toma T, Kim HC (2010). Molecular phylogenetics of *Aedes japonicus*, a disease vector that recently invaded western Europe, North America, and the Hawaiian islands. J Med Entomol..

[12] Kaufman MG, Fonseca DM (2014). Invasion biology of *Aedes japonicus japonicus* (Diptera: Culicidae). Annu Rev Entomol..

[13] Koban MB, Kampen H, Scheuch DE, Frueh L, Kuhlisch C, Janssen N (2019). The Asian bush mosquito *Aedes japonicus japonicus* (Diptera: Culicidae) in Europe, 17 years after its first detection, with a focus on monitoring methods. Parasit Vectors.

[14] Eritja R, Ruiz-Arrondo I, Delacour-Estrella S, Schaffner F, Álvarez-Chachero J, Bengoa M (2019). First detection of *Aedes japonicus* in Spain: an unexpected finding triggered by citizen science. Parasit Vectors.

[15] Janssen N, Graovac N, Vignjević G, Sudarić Bogojević M, Turić N, Klobučar A (2020). Rapid spread and population genetics of *Aedes japonicus* (Diptera: Culicidae) in southeastern Europe (Croatia, Bosnia and Herzegovina, Serbia). PLoS ONE.

[16] Schaffner F, Kaufmann C, Hegglin D, Mathis A (2009). The invasive mosquito *Aedes japonicus* in Central Europe. Med Vet Entomol..

[17] Zielke DE, Ibáñez-Justicia A, Kalan K, Merdić E, Kampen H, Werner D (2015). Recently discovered *Aedes japonicus japonicus* (Diptera: Culicidae) populations in the Netherlands and northern Germany resulted from a new introduction event and from a split from an existing population. Parasit Vectors.

[18] Fonseca DM, Widdel AK, Hutchinson M, Spichiger SE, Kramer LD (2010). Fine-scale spatial and temporal population genetics of *Aedes japonicus*, a new US mosquito, reveal multiple introductions. Mol Ecol..

[19] Werner D, Kampen H (2013). The further spread of *Aedes japonicus japonicus* (Diptera, Culicidae) towards northern Germany. Parasitol Res..

[20] Scott JJ. The ecology of the exotic mosquito *Ochlerotatus* (*Finlaya*) *japonicus japonicus* (Theobald 1901) (Diptera: Culicidae) and an examination of its role in the West Nile virus cycle in New Jersey. PhD thesis. New Brunswick: Rutgers University; 2003.

[21] Reuss F, Wieser A, Niamir A, Bálint M, Kuch U, Pfenninger M (2018). Thermal experiments with the Asian bush mosquito (*Aedes japonicus japonicus*) (Diptera: Culicidae) and implications for its distribution in Germany. Parasit Vectors.

[22] Andreadis TG, Wolfe RJ (2010). Evidence for reduction of native mosquitoes with increased expansion of invasive *Ochlerotatus japonicus japonicus* (Diptera: Culicidae) in the northeastern United States. J Med Entomol..

[23] Byrd BD, Sither CB, Goggins JA, Kunze-Garcia S, Pesko KN, Bustamante DM (2019). Aquatic thermal conditions predict the presence of native and invasive rock pool *Aedes* (Diptera: Culicidae) in the southern Appalachians, USA. J Vector Ecol..

[24] Chaves LF, Moji K (2018). Density dependence, landscape, and weather impacts on aquatic *Aedes japonicus japonicus* (Diptera: Culicidae) abundance along an urban altitudinal gradient. J Med Entomol..

[25] Iriarte WLZ, Tsuda Y, Wada Y, Takagi M (1991). Distribution of mosquitoes on a hill of Nagasaki City, with emphasis on the distance from human dwellings. Trop Med..

[26] Bartlett-Healy K, Unlu I, Obenauer P, Hughes T, Healy S, Crepeau T (2012). Larval mosquito habitat utilization and community dynamics of *Aedes albopictus* and *Aedes japonicus* (Diptera: Culicidae). J Med Entomol..

[27] Becker N, Pluskota B, Oo T, Huber K. Untersuchungen zur Einschleppung, Ausbreitung und Bekämpfung des japanischen Buschmoskitos (*Ochlerotatus japonicus*). Research Report KLIMOPASS. Karlsruhe: LUBW Baden-Württemberg State Institute for the Environment, Survey and Nature Conservation; 2014. https://pudi.lubw.de/detailseite/-/publication/37370-Buschmoskito_in_Baden-W%C3%BCrttemberg.pdf. Accessed 13 Sept 2020.

[28] Murrell EG, Noden BH, Juliano SA (2015). Contributions of temporal segregation, oviposition choice, and non-additive effects of competitors to invasion success of *Aedes japonicus* (Diptera: Culicidae) in North America. Biol Invasions..

[29] Andreadis TG, Anderson JF, Munstermann LE, Wolfe RJ, Florin DA (2001). Discovery, distribution, and abundance of the newly introduced mosquito *Ochlerotatus japonicus* (Diptera: Culicidae) in Connecticut, USA. J Med Entomol..

[30] Pulliam HR (1988). Sources, sinks, and population regulation. Am Nat..

[31] Delibes M, Gaona P, Ferreras P (2001). Effects of an attractive sink leading to maladaptive habitat selection. Am Nat..

[32] Sáringer-Kenyeres M, Bauer N, Kenyeres Z (2020). Active dispersion, habitat requirements and human biting behaviour of the invasive mosquito *Aedes japonicus japonicus* (Theobald, 1901) in Hungary. Parasitol Res..

[33] Peus F (1929). Beiträge zur Faunistik und Ökologie der einheimischen Culiciden. Zeitschr Desinfekt.

[34] Yates MG (1979). The biology of the tree-hole breeding mosquito *Aedes geniculatus* (Olivier) (Diptera: Culicidae) in southern England. Bull Entomol Res..

[35] Dekoninck W, Hendrickx F, Bortel WV, Versteirt V, Coosemans M, Damiens D (2011). Human-induced expanded distribution of *Anopheles plumbeus*, experimental vector of West Nile virus and a potential vector of human malaria in Belgium. J Med Entomol..

[36] Gomez-Cova CJ. Ecological studies on container-breeding mosquitoes *Aedes geniculatus* (Olivier) and *Aedes aegypti* (L.). PhD thesis. London: University of London; 1977.

[37] Reiskind MH, Griffin RH, Janairo MS, Hopperstad KA (2017). Mosquitoes of field and forest: the scale of habitat segregation in a diverse mosquito assemblage. Med Vet Entomol..

[38] Kerkow A, Wieland R, Koban MB, Hölker F, Jeschke JM, Werner D (2019). What makes the Asian bush mosquito *Aedes japonicus japonicus* feel comfortable in Germany? A fuzzy modelling approach. Parasit Vectors.

[39] Federal State North Rhine-Westphalia. Digitales Basis-Landschaftsmodell. 2018. https://www.opengeodata.nrw.de/produkte/geobasis/lm/basis-dlm/. Accessed 13 Sep 2020.

[40] European Centre for Disease Prevention and Control (2012). Guidelines for the surveillance of invasive mosquitoes in Europe.

[41] Becker N, Petric D, Zgomba M, Boase C, Madon M, Dahl C (2010). Mosquitoes and their control.

[42] Schaffner F, Angel G, Geoffroy B, Hervy J, Rhaiem A, Brunhes J. The mosquitoes of Europe. An identification and training programme (CD-ROM). Montpellier, France; 2001.

[43] Rudolf M, Czajka C, Börstler J, Melaun C, Jöst H, von Thien H (2013). First nationwide surveillance of *Culex pipiens* complex and *Culex torrentium* mosquitoes demonstrated the presence of *Culex pipiens* biotype *pipiens/molestus* hybrids in Germany. PLoS ONE.

[44] Connor M, Monroe W (1923). *Stegomyia* indices and their value in yellow fever control. Am J Trop Med Hyg..

[45] Focks DA (2003). A review of entomological sampling methods and indicators for dengue vectors.

[46] Benjamini Y, Yekutieli D (2001). The control of the false discovery rate in multiple testing under dependency. Ann Stat..

[47] R Core Team. R: a language and environment for statistical computing. 2019. https://www.R-project.org. Accessed 03 July 2019.

[48] R Core Team. foreign: read data stored by 'Minitab', 'S', 'SAS', 'SPSS', 'Stata', 'Systat', 'Weka', 'dBase', …. 2018. https://CRAN.R-project.org/package=foreign. Accessed 03 July 2019.

[49] Wickham H, Bryan J. readxl: read Excel files. 2019. https://CRAN.R-project.org/package=readxl. Accessed 03 July 2019.

[50] Mangiafico S. rcompanion: functions to support extension education program evaluation. 2019. https://CRAN.R-project.org/package=rcompanion. Accessed 31 Oct 2019.

[51] Oksanen J, Blanchet FG, Kindt R, Legendre P, McGlinn D, Minchin PR, et al. vegan: community ecology package, R package version 2.5-5. 2019. https://CRAN.R-project.org/package=vegan. Accessed 03 July 2019.

[52] Ter Braak CJ (1987). The analysis of vegetation-environment relationships by canonical correspondence analysis. Vegetation.

[53] Zeileis A, Kleiber C, Jackman S (2008). Regression models for count data in R. J Stat Softw..

[54] Kleiber C, Zeileis A (2016). Visualizing count data regressions using rootograms. Am Stat..

[55] Nagelkerke NJ (1991). A note on a general definition of the coefficient of determination. Biometrika.

[56] Fox J, Weisberg S (2018). An R companion to applied regression.

[57] Zeileis A, Kleiber C. countreg: count data regression. 2018. https://R-Forge.R-project.org/projects/countreg/. Accessed 31 Oct 2019.

[58] Wilke CO. cowplot: streamlined plot theme and plot annotations for ‘ggplot2’. 2019. https://CRAN.R-project.org/package=cowplot. Accessed 31 Oct 2019.

[59] Wickham H (2016). ggplot2: elegant graphics for data analysis.

[60] Bartón K. MuMIn: multi-Model Inference. R package version 1.43.6. 2019. https://CRAN.R-project.org/package=MuMIn. Accessed 09 Dec 2019.

[61] Jackman S. pscl: classes and methods for R developed in the political science computational laboratory. 2017. https://github.com/atahk/pscl/. Accessed 31 Oct 2019.

[62] Deutscher Wetterdienst. Aktuelle Pressemitteilungen. 2018. https://www.dwd.de/DE/presse/pressemitteilungen/pressemitteilungen_archiv_2018_node.html. Accessed 13 Sept 2020.

[63] Zielke DE, Walther D, Kampen H (2016). Newly discovered population of *Aedes japonicus japonicus* (Diptera: Culicidae) in Upper Bavaria, Germany, and Salzburg, Austria, is closely related to the Austrian/Slovenian bush mosquito population. Parasit Vectors.

[64] Walther D, Kampen H (2017). The citizen science project 'Mueckenatlas' helps monitor the distribution and spread of invasive mosquito species in Germany. J Med Entomol.

[65] Kampen H, Schuhbauer A, Walther D (2017). Emerging mosquito species in Germany—a synopsis after 6 years of mosquito monitoring (2011–2016). Parasitol Res..

[66] Beck HE, Zimmermann NE, McVicar TR, Vergopolan N, Berg A, Wood EF (2018). Present and future Köppen-Geiger climate classification maps at 1-km resolution. Sci Data..

[67] Bisanzio D, Giacobini M, Bertolotti L, Mosca A, Balbo L, Kitron U (2011). Spatio-temporal patterns of distribution of West Nile virus vectors in eastern Piedmont Region, Italy. Parasit Vectors.

[68] Nihei N, Komagata O, Mochizuki K-I, Kobayashi M (2014). Geospatial analysis of invasion of the Asian tiger mosquito *Aedes albopictus*: competition with *Aedes japonicus japonicus* in its northern limit area in Japan. Geospat Health..

[69] Schmidt M, Lischeid G, Nendel C (2019). Microclimate and matter dynamics in transition zones of forest to arable land. Agric For Meteorol..

[70] Verdonschot PFM, Besse-Lototskaya AA (2014). Flight distance of mosquitoes (Culicidae): a metadata analysis to support the management of barrier zones around rewetted and newly constructed wetlands. Limnologica..

[71] Gomes B, Sousa CA, Vicente JL, Pinho L, Calderón I, Arez E (2013). Feeding patterns of *molestus* and *pipiens* forms of *Culex pipiens* (Diptera: Culicidae) in a region of high hybridization. Parasit Vectors.

[72] Harbach RE, Harrison BA, Gad AM (1984). *Culex* (*Culex*) *molestus* Forskal (Diptera: Culicidae): neotype designation, description, variation, and taxonomic status. Proc Entomol Soc Wash..

[73] Fish D, Carpenter SR (1982). Leaf litter and larval mosquito dynamics in tree-hole ecosystems. Ecology.

[74] David J-P, Rey D, Pautou M-P, Meyran J-C (2000). Differential toxicity of leaf litter to dipteran larvae of mosquito developmental sites. J Invertebr Pathol..

[75] Vezzani D, Albicócco AP (2009). The effect of shade on the container index and pupal productivity of the mosquitoes *Aedes aegypti* and *Culex pipiens* breeding in artificial containers. Med Vet Entomol..

[76] Scheffers BR, Edwards DP, Diesmos A, Williams SE, Evans TA (2014). Microhabitats reduce animal's exposure to climate extremes. Glob Chang Biol..

[77] Müller R, Knautz T, Vollroth S, Berger R, Kreß A, Reuss F (2018). Larval superiority of *Culex pipiens* to *Aedes albopictus* in a replacement series experiment: prospects for coexistence in Germany. Parasit Vectors.

[78] Gjullin C, Hegarty C, Bollen W (1941). The necessity of a low oxygen concentration for the hatching of *Aedes* mosquito eggs. J Cell Physiol..

[79] Tippelt L, Werner D, Kampen H (2019). Tolerance of three *Aedes albopictus* strains (Diptera: Culicidae) from different geographical origins towards winter temperatures under field conditions in northern Germany. PLoS ONE.

[80] Ansari M, Singh K, Brooks G, Malhotra P, Vaidyanathan V (1977). The development of procedures and techniques for mass rearing of *Aedes aegypti*. Indian J Med Res..

[81] Zheng M-L, Zhang D-J, Damiens DD, Lees RS, Gilles JRL (2015). Standard operating procedures for standardized mass rearing of the dengue and chikungunya vectors *Aedes aegypti* and *Aedes albopictus* (Diptera: Culicidae). II. Egg storage and hatching. Parasites Vectors.

[82] Montarsi F, Martini S, Michelutti A, Da Rold G, Mazzucato M, Qualizza D (2019). The invasive mosquito *Aedes japonicus japonicus* is spreading in northeastern Italy. Parasit Vectors.

[83] Kampen H, Walther D. Vector potential of mosquito species (Diptera: Culicidae) occurring in Central Europe. In: Benelli G, Mehlhorn H, editors. Mosquito-borne diseases—implications for public health: Parasitology Research Monographs, vol. 10. 2018. p. 41-68.

[84] Kampen H, Holicki CM, Ziegler U, Groschup MH, Tews BA, Werner D (2020). West Nile virus mosquito vectors (Diptera: Culicidae) in Germany. Viruses..

[85] Chambers JM, Cleveland WS, Kleiner B, Tukey PA (1983). Graphical methods for data analysis.

