# Supplementary material for: Oviposition of Aedes japonicus japonicus (Diptera: Culicidae) and associated native species in relation to season, temperature and land use in western Germany
Source: Parasit Vectors. 2020 Dec 17;13:623. doi: 10.1186/s13071-020-04461-z (PMC7744736; doi:10.1186/s13071-020-04461-z)
Supplement: Supplementary file 1 — Additional file 1: Fig. S1. Study site 2017 at Dormagen with trap locations as related to land use type. Fig. S2. Study site 2017 at Alfter with trap locations as related to land use type. Due to logistical reasons, two transects overlap. Fig. S3. Heatmaps showing occurrence of mosquito taxa [Aedes japonicus japonicus (a), Culex pipiens s.l. (b), Anopheles plumbeus (c); mean positive ovitraps per trap location] in relation to trap location. F100 Forest, 100 m from the transition zone; F10 forest, 10 m from the transition zone; F/S transition zone; S10 settlement, 10 m from the transition zone; S100 settlement, 100 m from the transition zone. For the number of analysable samplings, see Fig. 3. Fig. S4. Rootograms of the model with different distribution types. Akaike information criterion (AIC) values: Gaussian = 498.9, Poisson = 538.4, zero-inflated = 483.2, negative binomial = 467.5. Table S1. List of traps positive for species and species combinations at trap locations in different land use types. C Cx. pipiens s.l., J Ae. japonicus japonicus, G Ae. geniculatus, P An. plumbeus, A arable land, S settlement area, F forest. Numbers in the description of the trap location indicate distance (m) to the transition zone, which is marked by a diagonal slash (e.g. A/F indicates arable land–forest transition zone). Species combinations represent numbers of traps with the specified taxa in the same trap. Table S2. Output of Fisher’s exact test (P-values) for the comparison of sampling dates by numbers of traps positive for Ae. japonicus japonicus. * P < 0.0001 Table S3. Land use-related oviposition in traps in Alfter and Dormagen 2017. For abbreviations, see Table S1. The mean of the portion of positive traps was tested between transects (different uppercase letters indicate significant difference) and trap locations (different lowercase letters indicate significant difference) according to Fisher’s exact test [file 13071_2020_4461_MOESM1_ESM.pdf]

Additional file 1

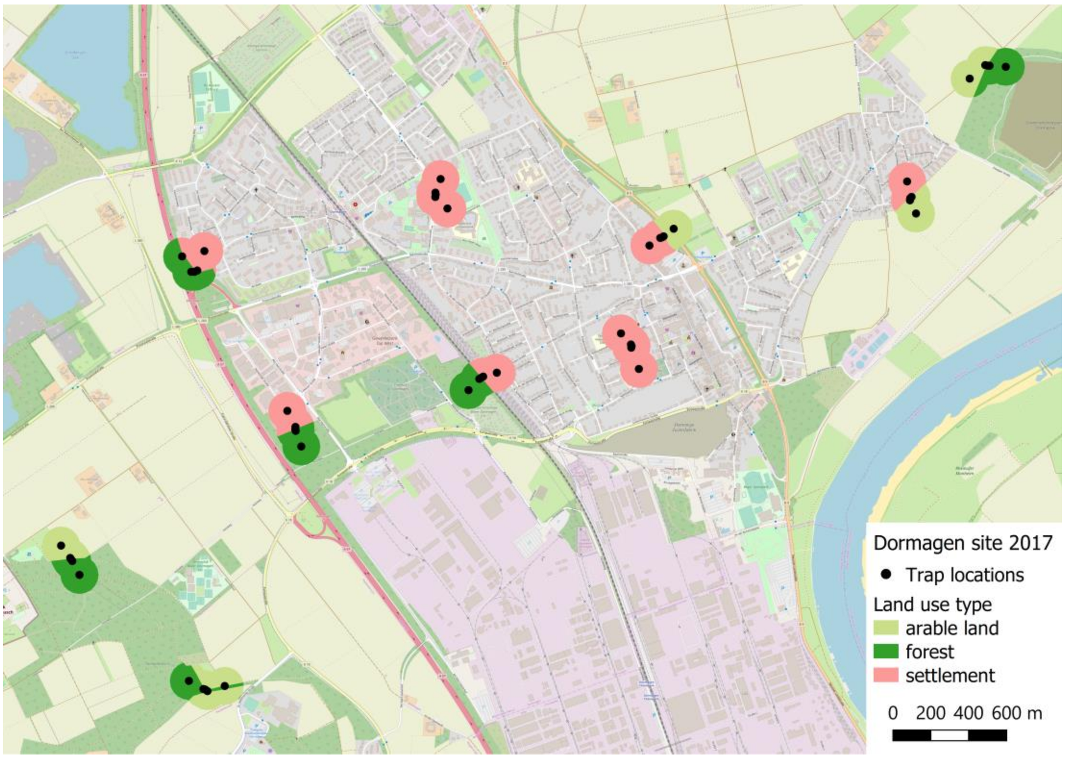

Figure S1: Study site 2017 at Dormagen with trap locations as related to land use type.

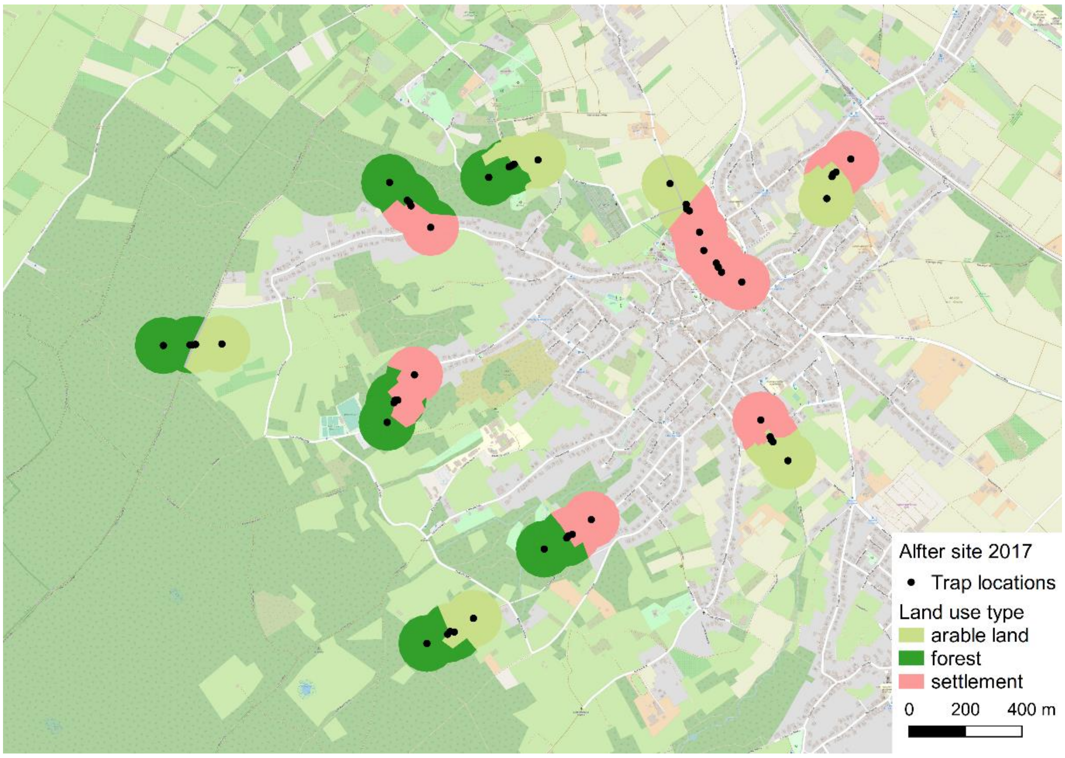

Figure S2: Study site 2017 at Alfter with trap locations as related to land use type. Due to logistical reasons, two transects overlap.

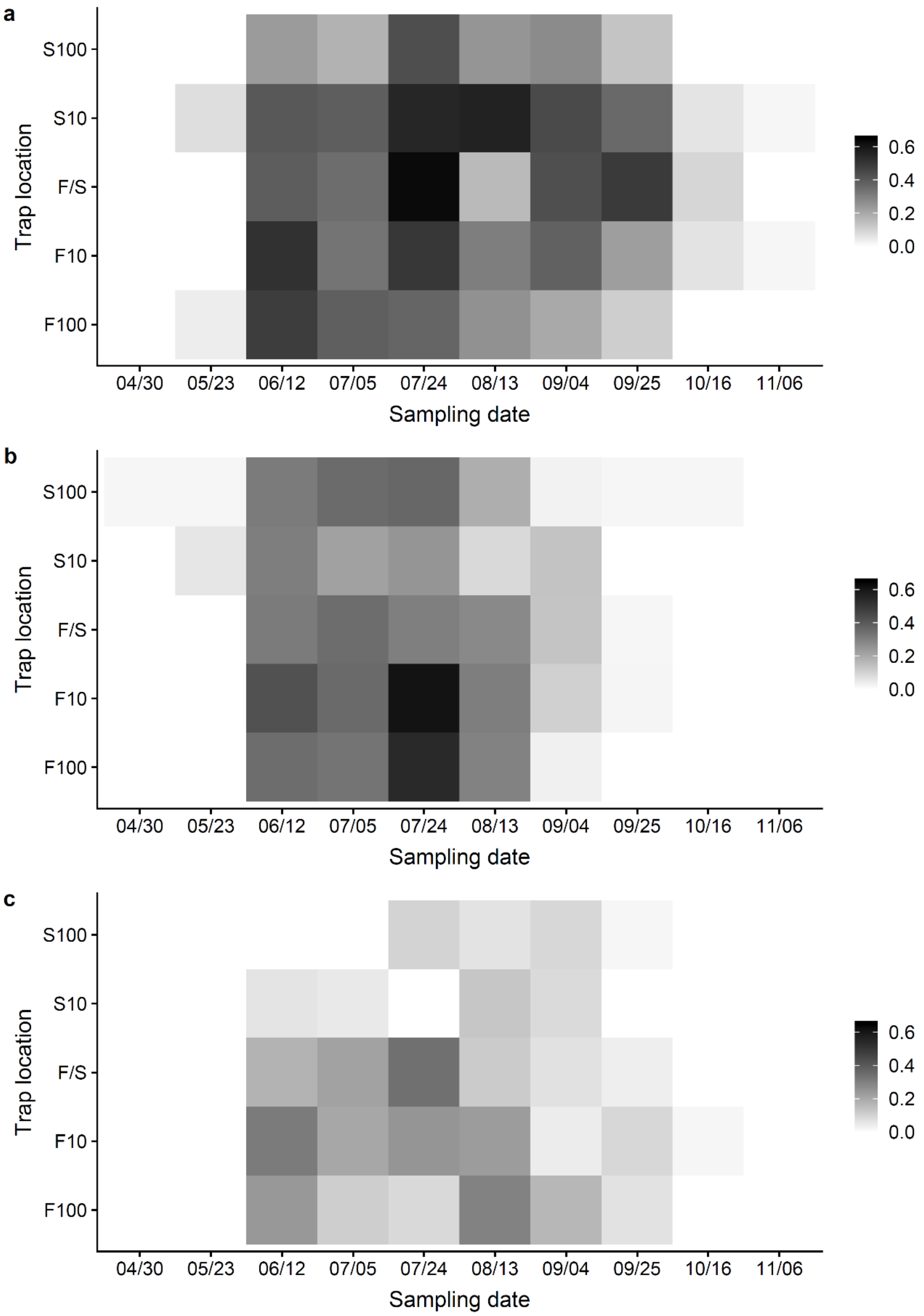

Figure S3: Heatmaps showing occurrence of mosquito taxa [*Ae. japonicus japonicus* (*a*), *Cx. pipiens* s.l. (*b*), *An. plumbeus* (*c*); mean ovitraps positive per trap location] in relation to trap location. *F100* Forest, 100 m distance to the transition zone; *F10* forest, 10 m distance to the transition zone; *F/S* transition zone; *S10* settlement, 10 m distance to the transition zone; *S100* settlement, 100 m distance to the transition zone. For the number of analysable samplings see Fig. 3.

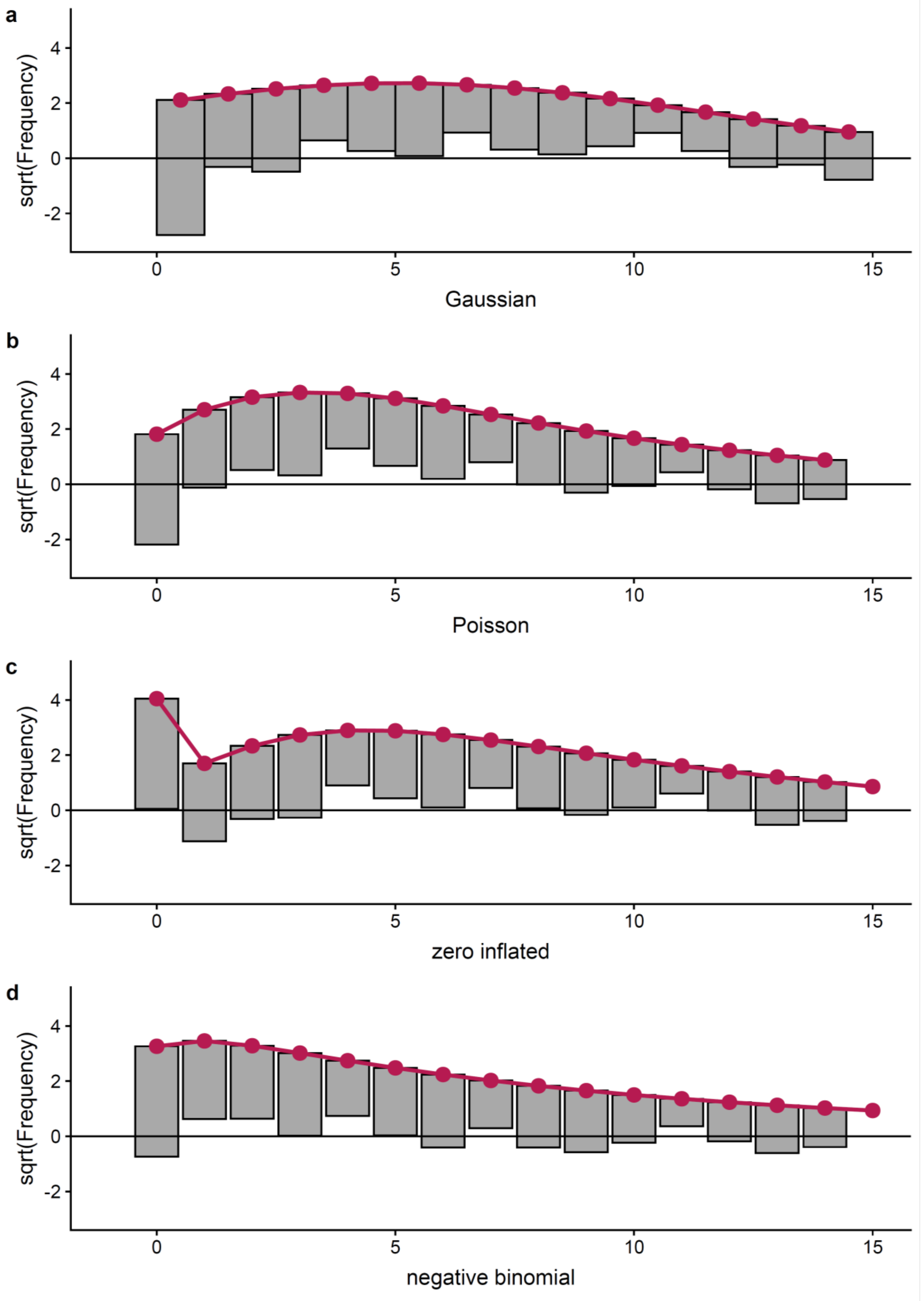

Figure S4: Rootograms of the model with different distribution types. AIC values: Gaussian = 498.9, Poisson = 538.4, zero-inflated = 483.2, negative binomial = 467.5

Table S1: List of positive traps of species and species combinations at trap locations in different land use types. *Abbreviations*: *C Cx. pipiens* s.l., *J Ae. japonicus japonicus*, *G Ae. geniculatus*, *P An. plumbeus*, *A* arable land, *S* settlement area, *F* forest. *Numbers in the description of the trap location* indicate distance (m) to the transition zone, which is marked by a *diagonal slash* (e. g. *A/F* indicates transition zone arable land–forest). *Species combinations* represent numbers of traps with the specified taxa in the same trap.

Year	Transect	Trap loc.	C	J	G	P	C+J	J+G	G+P	C+G	C+P	J+P	C+J+G	J+G+P	C+G+P	C+J+P	all
2017	A-F	A100	5	3	0	0	2	0	0	0	0	0	0	0	0	0	0
2017	A-F	A10	24	10	3	3	4	0	0	0	2	0	1	0	0	0	1
2017	A-F	A/F	7	8	3	4	0	0	0	0	0	1	0	1	0	1	0
2017	A-F	F10	15	4	4	8	0	1	1	2	2	1	0	0	0	2	0
2017	A-F	F100	10	9	9	8	0	2	0	1	3	0	0	1	0	2	0
2017	A-S	A100	7	2	0	0	0	0	0	0	0	0	0	0	0	0	0
2017	A-S	A10	12	0	1	2	0	0	0	0	0	0	0	0	1	0	0
2017	A-S	A/S	8	0	0	0	0	0	0	0	0	0	0	0	0	0	0
2017	A-S	S10	10	0	0	0	0	0	0	0	0	0	0	0	0	0	0
2017	A-S	S100	7	1	0	1	0	0	0	0	0	0	0	0	0	0	0
2017	F-S	F100	18	6	4	3	1	0	0	1	0	0	0	0	1	2	0
2017	F-S	F10	11	14	9	9	2	2	0	3	1	4	0	2	0	1	0
2017	F-S	F/S	17	24	1	9	7	1	0	0	4	4	0	0	0	1	0
2017	F-S	S10	16	13	2	0	6	1	0	0	0	0	0	0	0	0	0
2017	F-S	S100	10	3	1	0	2	0	0	1	0	0	0	0	0	0	0
2017	S	S	22	0	1	0	0	0	0	1	0	0	0	0	0	0	0
sum 2017			199	97	38	47	24	7	1	9	12	10	1	4	2	9	1
2018	F-S	F100	59	73	11	36	16	3	0	2	4	20	0	0	0	4	0
2018	F-S	F10	69	95	4	44	19	1	0	0	7	18	0	2	0	12	0
2018	F-S	F/S	68	110	4	38	17	3	0	0	10	12	0	0	0	10	0
2018	F-S	S10	44	113	0	11	19	0	0	0	2	4	0	0	0	2	0
2018	F-S	S100	45	50	0	8	13	0	0	0	1	4	0	0	0	1	0
sum 2018			285	441	19	137	84	7	0	2	24	58	0	2	0	29	0

Table S2. Output of Fisher's exact test (*P*-values) regarding the comparison of sampling dates by numbers of traps positive for *Ae. japonicus japonicus*. * $P < 0.0001$

Comparison	*P*
04/30 : 05/23	0.67
04/30 : 06/12	<0.0001*
04/30 : 07/05	<0.0001*
04/30 : 07/24	<0.0001*
04/30 : 08/13	<0.0001*
04/30 : 09/04	<0.0001*
04/30 : 09/25	<0.0001*
04/30 : 10/16	0.01
04/30 : 11/06	1.00
05/23 : 06/12	<0.0001*
05/23 : 07/05	<0.0001*
05/23 : 07/24	<0.0001*
05/23 : 08/13	<0.0001*
05/23 : 09/04	<0.0001*
05/23 : 09/25	<0.0001*
05/23 : 10/16	1.00
05/23 : 11/06	1.00
06/12 : 07/05	1.00
06/12 : 07/24	1.00
06/12 : 08/13	0.36
06/12 : 09/04	1.00
06/12 : 09/25	0.04
06/12 : 10/16	<0.0001*
06/12 : 11/06	<0.0001*
07/05 : 07/24	0.12
07/05 : 08/13	1.00
07/05 : 09/04	1.00
07/05 : 09/25	1.00
07/05 : 10/16	<0.0001*
07/05 : 11/06	<0.0001*
07/24 : 08/13	0.01
07/24 : 09/04	0.37
07/24 : 09/25	0.0003
07/24 : 10/16	<0.0001*
07/24 : 11/06	<0.0001*
08/13 : 09/04	1.00
08/13 : 09/25	1.00
08/13 : 10/16	<0.0001*
08/13 : 11/06	<0.0001*
09/04 : 09/25	1.00
09/04 : 10/16	<0.0001*
09/04 : 11/06	<0.0001*
09/25 : 10/16	<0.0001*
09/25 : 11/06	<0.0001*
10/16 : 11/06	0.48

Table S3. Land use related oviposition in traps in Alfter and Dormagen 2017. For abbreviations of land use types (Transect, Trap location) see Table S1. The mean of the portion of traps positive was tested between transects (*different uppercase letters* indicate significant difference) and trap locations (*different lowercase letters* indicate significant difference) by Fisher's exact test.

		Analysable samplings		*Ae. japonicus japonicus*			*Cx. pipiens* s.l.		*An. plumbeus*		*Ae. geniculatus*	
Transect	Trap location	Alfter	Dormagen	Alfter	Dormagen	$P < 0.05$	Alfter	Dormagen	Alfter	Dormagen	Alfter	Dormagen
A-S	A100	16	9	0.125	0	abc	0.438	0	0	0	0	0
	A10	22	6	0	0	ab	0.545	0	0.091	0	0.045	0
	A/S	19	10	0	0	ab	0.211	0.4	0	0	0	0
	S10	21	13	0	0	ab	0.286	0.308	0	0	0	0
	S100	20	10	0.05	0.1	abc	0.2	0.3	0	0	0	0
	Total	98	48	0.031	0.021	C	0.337	0.229	0.02	0	0.01	0
A-F	A100	16	15	0.188	0	abc	0.25	0.067	0	0	0	0
	A10	24	22	0.333	0.091	acd	0.583	0.455	0.125	0	0.125	0
	A/F	22	19	0.364	0	acd	0.136	0.211	0.182	0	0.045	0.053
	F10	24	23	0.125	0.043	abc	0.375	0.261	0.292	0.043	0.042	0.087
	F100	27	20	0.333	0	acd	0.259	0.15	0.259	0.05	0.185	0.15
	Total	113	99	0.274	0.03	A	0.327	0.242	0.186	0.02	0.088	0.061
F-S	F100	27	16	0.222	0	abc	0.37	0.5	0.111	0	0.111	0.063
	F10	26	22	0.462	0.091	cd	0.269	0.182	0.308	0.045	0.115	0.273
	F/S	29	18	0.655	0.278	d	0.414	0.278	0.31	0	0.034	0
	S10	26	16	0.5	0	cd	0.462	0.25	0	0	0.038	0
	S100	11	15	0.091	0.133	abc	0.273	0.467	0	0	0	0.067
	Total	119	87	0.429	0.103	B	0.37	0.322	0.168	0.011	0.067	0.092
S	Total	22	42	0	0	b C	0.136	0.452	0	0	0	0.024
